# Diabetic Foot Ulcers and Their Surgical Management: Our Experience at Hayatabad Medical Complex, Peshawar

**DOI:** 10.7759/cureus.48073

**Published:** 2023-10-31

**Authors:** Rashid Aslam, Khalid Usman, Tahir Ghaffar

**Affiliations:** 1 Department of General Surgery, Hayatabad Medical Complex, Peshawar, PAK; 2 Department of Diabetes and Endocrinology, Hayatabad Medical Complex, Peshawar, PAK

**Keywords:** diabetic foot ulcers, diabetic foot ulcers management, wagner classification, prevalence, amputation, diabetes, ulcers

## Abstract

Background: Surgeons face a therapeutic challenge while treating diabetic foot ulcers (DFUs), particularly in underdeveloped nations with limited healthcare resources and a high proportion of patients who arrive at medical institutions with advanced foot ulcers.

Objective: To assess the effectiveness of treatment in patients with DFUs and to demonstrate how early surgical intervention and appropriate bedside medical care can improve results.

Material and methods: This prospective study was carried out at Hayatabad Medical Complex, Peshawar, Pakistan, to assess how DFUs changed over a period between November 2021 and December 2022 at the wards and at the outpatient department of endocrinology and general surgery. A diabetic patient's foot is first screened for ulceration in the endocrinology department, and only those with active ulcers are referred to the surgical department.

Results: According to the Wagner classification, there were six (13.6%) cases in grade I, 11 (25%) in grade 2, 10 (22.7%) in grade 3, 13 (29.5%) in grade 4, and four (9%) in grade 5. Among comorbidities, a family history of diabetes mellitus was noted in 25 (56.8%) patients, followed by tobacco chewing and alcohol in 10 (22.7%) and hypertension in nine (20.4%) patients.

Conclusion: Diabetes foot ulceration is the most common reason for non-traumatic lower limb amputation in people with diabetes mellitus and is a significant cause of morbidity and mortality.

## Introduction

A diabetic foot is a serious medical condition in which poor control of blood sugar levels can lead to serious foot complications, such as ulcers, infections, and even amputations [1]. In Pakistan, the prevalence of diabetic foot is alarmingly high [2]. Diabetic foot ulcers (DFUs) present a significant health challenge in Pakistan, driven by a combination of high diabetes prevalence, complications, and limited healthcare access. Pakistan ranks among the countries with the highest diabetes prevalence, with an estimated 33 million adults affected in 2021 [3]. Diabetic foot ulcers, a severe diabetes complication, are common and often lead to amputations. High amputation rates further compound the healthcare and economic burdens. Moreover, limited access to healthcare resources and strained infrastructure exacerbate the problem. To address this major health issue, comprehensive efforts are needed to improve diabetes management, enhance healthcare infrastructure, raise awareness, and ensure effective prevention and treatment of DFUs [4]. Diabetic foot ulcers are more common among people with type 2 diabetes and those living in rural areas of Pakistan. It is also more prevalent among men than women. The risk factors for DFUs include poor glycemic control, peripheral neuropathy, peripheral vascular disease, and a high body mass index [5].

Diabetic foot ulcers are a serious health problem in Pakistan, as they can lead to amputation and even death. Early detection and timely treatment are essential for preventing complications. It is important for people with diabetes to be aware of the risk of DFUs and to consult a doctor at the earliest signs of infection [6].

Diabetic foot ulcers can be difficult to treat due to their slow healing time. When individuals with diabetes experience impaired blood flow due to vascular issues, it can substantially slow down the healing process of DFUs. Treatment typically includes wound care, debridement, offloading, and infection control. Wound care involves keeping the wound clean and hydrated, as well as avoiding further trauma. Debridement involves removing necrotic tissue from the wound, which can help reduce infection. Offloading is the use of specialized shoes or casts to reduce weight on the ulcerated area, while infection control involves the use of antibiotics and other medications [7].

Diabetic foot ulcers can have serious consequences if left untreated, including amputation of the affected limb. To prevent this, it is important to practice proper foot care, check for signs of an ulcer, and seek medical attention if an ulcer is detected. With proper treatment, the majority of DFUs can be healed without the need for amputation [8].

Healthcare providers in Pakistan should also be aware of the high prevalence of DFUs and should take steps to diagnose and treat them early. This includes providing education on proper foot care, regular screening for foot infections, and prompt referral to specialists when needed.

## Materials and methods

After approval from the Hospital Research and Ethical Committee (reference # 1245-8), this prospective study was carried out at Hayatabad Medical Complex, Peshawar, Pakistan, to assess how DFUs changed over a period between November 2021 and December 2022 at the wards and at the outpatient department of endocrinology and general surgery. A diabetic patient's foot is first screened for ulceration in the endocrinology department, and only those with active ulcers are referred to the surgical department.

The study included all patients with DFUs who consented to participate, and those who met the inclusion criteria were enrolled consecutively. The study excluded patients with healed foot ulcerations. Diabetic patients who were found to have active foot ulceration were referred to the surgical wards or surgical operation theater (OT) for proper surgical management. The endocrinology ward was used to identify patients with foot ulcers at risk. Operating definitions of DFUs included induration, ulceration, change in color, or a breach in the normal skin on the foot that lasted for at least two weeks. A detailed history and physical examination were performed, including the length of diabetes, the type of diabetes (type I or II), the duration of foot ulcers and awareness of them, the mode of treatment received, previous foot care knowledge, previous history of healed foot ulcers, Wagner's classification [9], and the type of DFU (neuropathic, ischemic, or neuro-ischemic). Amputation at or proximal to the ankle joint was defined as a major lower limb amputation, while amputation distal to the ankle joint was classified as a minor lower limb amputation. Glycated hemoglobin (HbA1c) was used to measure glycemic control.

Wagner's classification was used to grade ulcers [9]. IBM SPSS software, version 23.0 (IBM Corp., Armonk, NY) and Microsoft Excel 2013 (Microsoft Corp., Redmond, WA) were used to collect and analyze the data. P-values less than 0.05 were considered significant.

## Results

A total of 44 patients were included, with an age range of 40 to 70 years and a mean of 55 years. Type 1 diabetes was found in 13 (29.5%) patients, while type 2 diabetes was found in 31 (70.5%) cases. Under the category of patients with type 1 diabetes, there were 11 (25%) males and 10 (22.7%) females, while for type 2 diabetes patients, this proportion was 14 (31.8%) and nine (20.4%), respectively. Within type 1 diabetes patients, five (11.4%) were 40-50 years old, five (11.4%) were 51-60 years old, and 13 (29.5%) were in the age group of 61-70 years old. Within type 2 diabetes patients, four (9.1%) were 40-50 years old, seven (15.9%) were 51-60 years old, and 10 (22.7%) were 61-70 years old, respectively (Table 1).

**Table 1:** Demographic details of the study participants

Demographics	Type 1 diabetes (n=13)	Type 2 diabetes (n=31)
Gender		
Male	11 (25%)	14 (31.8%)
Female	10 (22.7%)	9 (20.4%)
Age group		
40-50 years	5 (11.4%)	4 (9.1%)
51-60 years	5 (11.4%)	7 (15.9%)
61-70 years	13 (29.5%)	10 (22.7%)

According to the Wagner classification [9], there were six (13.6%) cases in grade I, 11 (25%) in grade 2, 10 (22.7%) in grade 3, 13 (29.5%) in grade 4, and four (9%) in grade 5. Among comorbidities, a family history of diabetes mellitus was noted in 25 (56.8%) patients, followed by tobacco chewing and alcohol (10 (22.7%)) and hypertension in nine (20.4%) patients. In terms of the duration of diabetes, 16 (36.4%) cases had been living with diabetes for over 10 years, 18 (40.9%) had a diabetes duration ranging from five to 10 years, five (11.4%) patients had been diagnosed with diabetes for less than five years, and five (11.4%) were diagnosed upon admission, indicating delayed identification of the condition. The HbA1c levels in the range of 5.7%-6.4% were documented in seven (15.9%) cases, while 6.5%-7% HbA1c levels were found in 16 (36.4%) cases. Notably, HbA1c levels above 7% were observed in the majority of cases, comprising 21 (47.7%) cases. Neuropathic ulcers were present in nine (20.4%) cases, ischemic ulcers in 12 (27.2%) cases, neuro-ischemic ulcers in 10 (22.7%) cases, and infectious ulcers in 13 (29.5%) cases. Regarding the duration of these foot ulcers, it was noted that 29 (65.9%) had ulcers persisting for over a month, while 15 (34.1%) had ulcers for less than a month, indicating variations in the chronicity of these wounds. The history of previously healed foot ulcers was documented in 21 (47.7%) cases, as shown in Table 2.

**Table 2:** Other characteristics of the study population

Characteristics	Frequency	Percentage
Wagner classification [9]		
Grade 1	6	13.6%
Grade 2	11	25%
Grade 3	10	22.7%
Grade 4	13	29.5%
Grade 5	4	9%
Comorbidity		
Family history	25	56.8%
Tobacco and alcohol use	10	22.7%
Hypertension	9	20.4%
Duration of diabetes (in years)		
<5	10	22.7%
5-10	18	40.9%
>10	16	36.4%
HbA1c level (%)		
5.7-6.4	7	15.9%
6.5-7	16	36.4%
>7	21	47.7%
Type of ulcer		
Neuropathic	9	20.4%
Ischemic	12	27.2%
Neuro-ischemic	10	22.7%
Infectious	13	29.5%
Duration of the ulcer		
Less than one month	15	34.1%
More than one month	29	65.9%

Swab culture results were positive for *Staphylococcus aureus* in 18 (40.9%) cases,* Klebsiella pneumoniae* in eight (18.1%) cases, *Pseudomonas aeruginosa* in seven (15.9%) cases, beta-hemolytic streptococci were isolated in five (11.3%) cases, and anaerobic cocci were found in six (13.6%) cases (Table 3).

**Table 3:** Positive swab culture results

Bacteria	Frequency	Percentage
Staphylococcus aureus	18	40.9%
Klebseilla pneumoniae	8	18.1%
Pseudomonas aeruginosa	7	15.9%
Beta-hemolytic streptococci	5	11.3%
Anaerobic cocci	6	13.6%

The most common presenting symptom was gangrene diabetic foot, seen in six patients (13.3%), five (11.4%) had diabetic foot cellulitis, four (9%) had abscesses, two (4.5%) had necrotizing fasciitis, and 27 (61.4%) had symptoms of DFUs (Table 4).

**Table 4:** Mode of presentation

Mode of presentation	Frequency	Percentage
Gangrenous diabetic foot	6	13.3%
Cellulitis	5	11.4%
Abscess	4	9%
Necrotizing fasciitis	2	4.5%
Ulcer	27	61.4%

Surgical interventions were carried out in 34 (77.3%) cases, while 10 (22.7%) cases were treated conservatively with daily dressing, and debridement in less severe cases where the wound was managed without the need for a surgical procedure and antibiotics. The most common surgical intervention was ray amputation in 13 (29.5%) cases, followed by incision and drainage in 12 (27.3%) cases, below-knee amputation in five (11.4%) cases, and above-knee amputation in four (9%) cases, respectively (Table 5).

**Table 5:** Different management options for diabetic foot ulcers

Management	Frequency	Percentage
Conservative (n=10)		
Dressing and debridement	10	22.7%
Surgical (n=34)		
Ray amputation	13	29.5%
Incision and drainage	12	27.3%
Below-knee amputation	5	11.4%
Above-knee amputation	4	9%

The most common postoperative wound complication was surgical site infection in nine (20.4%) cases, followed by recurrence in seven (15.9%) cases and deformity in four (9%) cases. Mortality was recorded in five (11.3%) cases, where two (4.5%) died due to renal failure (diabetes-related nephropathy and pre-existing kidney diseases), two (4.5%) due to myocardial infarction (MI), and one (2.3%) due to septicemia (Table 6).

**Table 6:** Postoperative complications, mortality, and its causes

	Frequency	Percentage
Wound complications		
Surgical site infection	9	20.4%
Recurrence	7	15.9%
Deformity	4	9%
Mortality and its causes		
Renal failure	2	4.5%
Myocardial infarction	2	4.5%
Septicemia	1	2.3%
Total mortality	5	11.3%

## Discussion

Infected foot ulcers can progress to deeper spaces and tissues, resulting in potentially disastrous results. Patients with diabetic foot infections may require foot amputation if not treated promptly and appropriately. In order to avoid contamination and optimize the identification of pathogens, specimens for culture should be obtained after wound debridement. Diabetic foot ulcers are more likely to develop in older diabetic patients [10].

Insensate feet are more likely to develop ulcers due to sensory neuropathy and impaired proprioception; this decreases the foot's ability to adapt to repetitive local stresses. In our study, a prevalence of 61.4% of diabetic foot ulcers was found, which was very high compared to the studies done in India and China, which noted almost 10%-14% of patients having diabetic foot ulcers [11]. It is possible that these differences are due to regional differences in the prevalence of diabetes mellitus and local operating risk factors. A variety of diabetes treatment centers offered different levels of quality in these studies. Since diabetes develops and progresses over time, this may suggest certain time-dependent risk factors that are common to all kinds of diabetes. There are also differences in the age at which diabetes begins on different continents.

The results of our study indicate that among the individuals included, males exhibited a higher prevalence of DFUs at 56.8%, a finding that aligns closely with the study by Lakhani et al., who reported a 56% prevalence [12]. *Staphylococcus aureus* (40.9%) was the most commonly isolated organism on swab culture, followed by *Klebsiella pneumoniae* (18.1%). These findings are quite similar to the findings of the study by Gohel et al. and Lakhani et al. [12,13].

The majority of amputations (50%) in our study were performed in cases of DFUs. Incision and drainage were done in 27.3% of cases, and debridement was done in 22.7% of cases. The results were comparable to those of Chalya et al., who observed almost 57% of amputations and 40% of debridement, and incision and drainage [14].

Healing of ulcers was achieved in 10 (22.7%) cases by wound debridement, excision of sloughs, and daily dressings. Other treatment methods include incision and drainage, ray amputation, below-knee amputations, and above-knee amputations. Nilsson et al. reported 87% successful limb salvages after repeated 'piecemeal' debridement and herbal drinks [15]. In order to optimize the wound environment and promote healing, saline-soaked gauze dressings are used; these dressings help to retain moisture and enhance the wound environment.

A mortality rate of 11.3% was observed in our study, which is higher than that reported by Muqim et al. and Bartus et al. [16,17]. Many of the patients in our study had advanced DFUs and sepsis when they were admitted to our hospital, resulting in multiple organ failure and death.

Our research has certain limitations. In our study, we used a small cohort and a single tertiary care hospital. Ideally, further studies should be conducted with large cohorts of patients from multiple hospitals in order to achieve better results.

## Conclusions

Foot ulcers in diabetic patients represent a significant burden, often leading to lower extremity amputation. Prioritizing diabetes education is crucial. As prevention outweighs the cure, effective glycemic control and education are paramount in reducing diabetic foot disease. Additionally, early presentation, prompt hospital admission, and tailored medical and surgical interventions based on the disease's severity can significantly enhance outcomes, ultimately mitigating the morbidity and mortality associated with diabetes.
